# Toward Highly Efficient Cancer Imaging and Therapy Using the Environment-Friendly Chitosan Nanoparticles and NIR Laser

**DOI:** 10.3390/bios9010028

**Published:** 2019-02-18

**Authors:** Hala S. Abuelmakarem, Mahmoud A. Sliem, Jala El-Azab, Moustafa M. A. Farghaly, Wafaa A. Ahmed

**Affiliations:** 1 System and Biomedical Engineering Department, The Higher Institute of Engineering, El Shorouk City, Cairo 11837, Egypt; mousfar@hotmail.com; 2 Department of Laser Applications in Metrology, Photochemistry and Agriculture (LAMPA), National Institute of Laser Enhanced Sciences (NILE), Cairo University, Giza 12613, Egypt; mahmoud.ashour@rub.de; 3 Department of Engineering Applications of Laser, National Institute of Laser Enhanced Sciences (NILES), Cairo University, Giza 12613, Egypt; jala@niles.edu.eg; 4 Cancer Biology Department, Biochemistry and Molecular Biology Unit, National Cancer Institute, Cairo University, Giza 11796, Egypt; drwafaaeg@yahoo.com

**Keywords:** nanoparticles, NIR, Chitosan, cytotoxicity, optical imaging

## Abstract

Chitosan-tripolyphosphate nanoparticles (C-TPP NPs) were synthesized to investigate their cytotoxicity against colon cancer cells (Caco2 cells) in the absence and the presence of a near-infrared (NIR) laser to evaluate their influence in cancer detection using the NIR laser and to evaluate the NIR laser on cancer treatment. The synthesized NPs were characterized by Fourier transform infrared (FT-IR) spectroscopy, dynamic light scattering (DLS), zeta potential (ZP), and transmission electronic microscope (TEM). The cytotoxicity was analyzed by the MTT test and the cell viability was assessed using the Trypan blue method. C-TPP NPs showed increased cytotoxicity and decreased cell viability against Caco2 cells. Upon laser exposure only, the cell viability decreased. The C-TPP NPs appeared to have a shining light on the cancerous cells which were photographed under the inverted microscope.

## 1. Introduction 

**Scheme 1:**
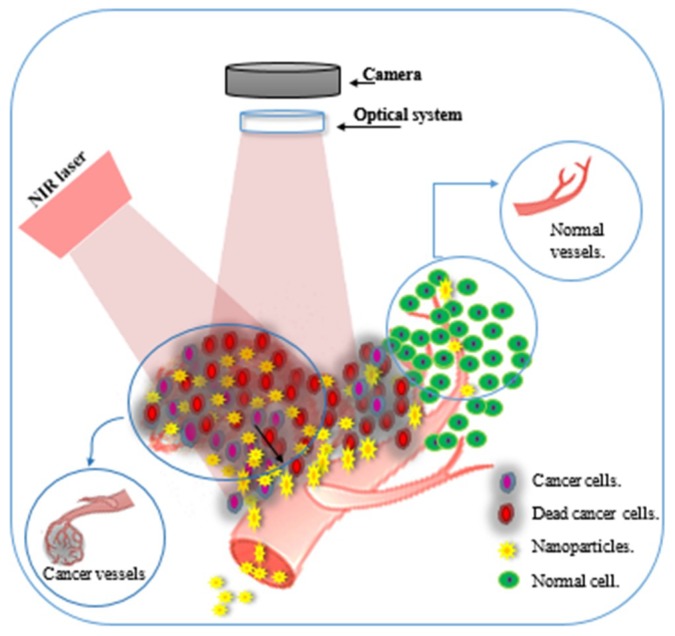
Schematic illustrates • the different structure between cancerous cells and normal cells, • the different structure between cancer blood vessels and normal blood vessels, • nanoparticles accumulation in cancerous tissue, • Near-infrared (NIR) has a maximum depth of penetration in the biological tissue.

Colon cancer is the third most common cancer diagnosed in the United States [1]. Colon cancer management depends on the stage of the tumor. The classic therapeutic strategies for most cancers depend on the stage and the degree of the tumor. Classical cancer diagnostic techniques are very expensive and have their limitations [2]. Optical imaging is a noninvasive and inexpensive technology. Optical imaging uses light to probe cellular and molecular function in cancer detection. Since cancerous cells have altered blood flow and are highly hypoxic, differential optical absorbances of oxy- and deoxyhemoglobin in the cancerous and normal cells have been used as an indicator for the cancer tissue (Scheme 1) [3]. Near-infrared (NIR) imaging is a powerful diagnostic method with the potential to serve as a nonionizing technique for sensitive, deep tissue diagnostic imaging due to its long wavelengths compared to ultraviolet (UV) [4,5]. Defining the tumor location by optical contrast agents allows the physician to better distinguish between normal and cancerous tissue [3]. Contrast-enhanced NIR imaging increases the sensitivity and the specificity of cancer detection. Nanoparticles used for NIR imaging such as NIR-emitting semiconductor quantum dots (QDs), resonant gold nanoshells, and dye-encapsulating nanoparticles have several limitations [4]. 

Chitosan is a natural product approved by both the food & drug administration (FDA) and European medicine agency (EMA) [6]. Chitosan culture system provides a platform for cancer therapy and imaging [7] due to its biocompatibility, physicochemical and functional properties [8]. Chitosan is an amino polysaccharide molecule. It has strongly positive electrostatic charge which attracts strongly the negatively charged molecules [9]. Chitosan nanoparticles (NPs) have photonic applications such as drug delivery, cell imaging, gene therapy, photothermal therapy, cancer diagnosis and cell imaging [7]. Chitosan NPs accumulate in cancerous tissue more than they do in normal tissues due to the enhanced permeability and retention (EPR) effect (Scheme 1) [10] and consequently are suitable for cancer management applications.

Previous research demonstrated the role of chitosan NPs in cancer management for both diagnosis and detection purposes, Yang et al. developed nano-composites from the alginate to physically complex with folic acid-modified chitosan for fluorescent endoscopic detection of colorectal cancer [11]. Chen et al. developed a probe using nanoparticles of miR-155 MB self-assembled with chitosan to detect the expression of miR-155 in lung cancer cells [12]. For the therapeutic purpose, Aruna et al. demonstrated chitosan NPs role in cancer treatment against many cancers [6]. In vitro, Loutfy et al. assayed the cytotoxicity of chitosan NPs against a liver cancer cell line (HepG2) [10]. Chitosan NPs are safe on the normal tissue as reported by Huang et al. who evaluated their cytotoxicity against A549 cells (adenocarcinomic human alveolar basal epithelial cells) [13].

The aim of this study is evaluating the cytotoxic effect of the C-TPP NPs in the presence and the absence of a NIR laser against colon carcinoma (Caco 2 cells) and to also observe their shining effect during NIR exposing. That will lead to develop imaging system based on modeling and simulating the process of photon transport in biological tissues.

## 2. Material and Methods

### 2.1. Material

Medium-molecular-weight chitosan (161.1 MW) with degree of deacetylation of 75% and TPP (Na_5_P_3_O_10_, molecular weight: 367.86) were purchased from Oxford company (Mumbai, India). Acetic acid was obtained from Merck. Diode Laser (660 nm, 10 mW/cm^2^, Mode: continuous) was purchased from the laser land company (Hubei, China).

### 2.2. Chitosan Nanoparticles Preparation 

**Scheme 2:**
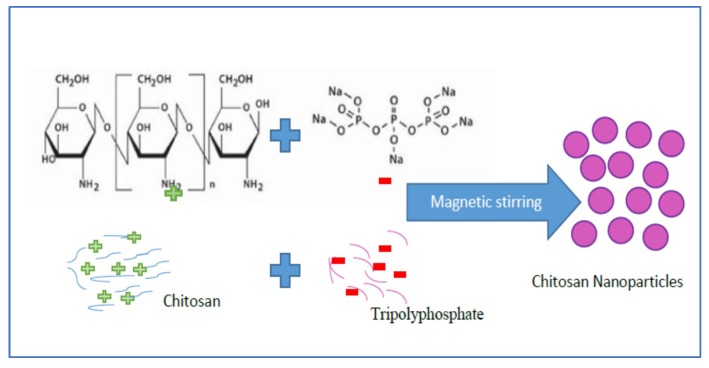
Schematic illustrates C-TPP NPs preparation.

The chitosan NPs (C-TPP NPs) was formed by the ionic gelation method (ionic crosslinking technology). That was based on the interaction between the positively charged amine group (chitosan) and negatively charged polyanions (tripolyphosphate (TPP)). Cationic solution of chitosan was obtained by dissolving 100 mg of chitosan powder in 1% diluted acetic acid (100 mL) under continuous magnetic stirring for 2 h at 2000 rpm. The anionic solution of TPP was obtained by dissolving TPP into the distilled water, then the TPP solution was dropped into a cationic solution of chitosan under magnetic stirring (Scheme 2). 

### 2.3. Physicochemical Characterization of the C-TPP NPs 

#### 2.3.1. Fourier Transform Infrared (FT-IR) Spectroscopy

Infrared spectra of chitosan and the C-TPP NPs were measured on a FT/IR-4100 type (A) instrument (JASCO, Tokyo, Japan) equipped with a triglycine sulfate detector (TGS) detector.

#### 2.3.2. Particle Size and Zeta Potential Measurements 

The hydrodynamic diameter and the surface charge of the C-TPP NPs were analyzed by dynamic light scattering (DLS) using ZS-ZEN (Malvern Instruments Co., Malvern, UK). The instrument measures the particle diameter size in the range from 0.6 to 6000 nm and the surface charge in the range from −200 to 200 mV. The samples were measured at a scattering angle of 173° at room temperature.

#### 2.3.3. Transmission Electron Microscope (TEM)

High resolution transmission electron microscope (HR-TEM, Tecnai G20, FEI, Eindhoven, Netherland) was used for the purpose of imaging, crystal structure revelation, elemental analysis qualitative and semi-quantitative analysis. Two different modes of imaging were employed; the first was, the bright field at an electron accelerating voltage of 200 kV using a lanthanum hexaboride (LaB6) electron source gun and diffraction pattern imaging. The second was an eagle CCD camera with (4k × 4k) image resolution that was used to acquire and collect transmitted electron images. TEM Imaging & Analysis (TIA) software was used for spectrum acquisition and analysis of EDX peaks.

The TEM was applied on two steps: first, the nanocomposite samples were pipetted up and down to be suspended; second, 2–5 μL drops of samples were mounted on carbon-coated 400-mesh copper grids and the specimens were left to dry for 2 min. The filter paper was used to remove the excess solution and to facilitate the settle down of particles on the grids.

### 2.4. Cytotoxicity Analysis by MTT Assay and Cancerous Cells Screening

The effect of the C-TPP NPs on cell viability was estimated using a 3-(4,5-dimethylthiazol-2-yl)-2,5-diphenyltetrazolium bromide (MTT) assay. Caco-2 cell line (colon cancer) was obtained from the National Cancer Institute, Cairo, Egypt. The cytotoxicity of the C-TPP NPs was evaluated against Caco-2 cell line in the absence and the presence of NIR. The cells were cultured in RPMI 1640 medium (Sigma-Aldrich, St Louis, MO, USA) and were seeded into 96-well plate and incubated. Briefly, after maintaining the cells in RPMI 1640 medium for 24 h, the medium of each well was removed, the cells were washed twice using 1.0 M phosphate-buffered saline (PBS; pH 7.4) at 25 °C, and the medium was replaced with C-TPP NPs in a range of concentrations (6.25–100) μg/mL. The cells were incubated at 37 °C in a 5% CO_2_ incubator for 24 h. Then, 10 mL of MTT solution (5 mg/1 mL of 1.0 M PBS pH 7.4) was added. Then, the cells incubated for 4 h at 37 °C in a 5% CO_2_ incubator. The medium was discarded and each well was supplemented with 100mL of dimethyl sulphoxide, mixed thoroughly using a pipette, and incubated in a dark room for 2 h. The absorbance (abs) of each well was read at 570 nm with a plate reader (SunriseTM; Tecan Group, Männedorf, Switzerland). The cell viability assay versus various concentrations was calculated using the below equation. GraphPad Prism 7 software (GraphPad Software, La Jolla, CA, USA) was used to determine the half-maximal inhibitory concentration (IC_50_) dose.

$$viabe\;cells\left(\%\right)=\frac{\left(abs_{sample}-abs_{blank}\right)}{\left(abs_{control\left(untreated\;cells\right)}-abs_{blank}\right)}\times 100$$

The morphological changes and the shining effect of C-TPP NPs were observed under the inverted microscope (Zeiss Axio Vert.A1; Zeiss, Gottingen, Germany) at 40_magnification. This observation was for the cells treated with the IC_50_ doses of C-TPP NPs in the presence and the absence of NIR. Also, the morphological change for the cells treated with the laser only (660 nm, 5 min) was observed. The cells were photographed using digital camera. Then, the cell viability was re-examined by trypan blue method. Besides, the cell viability for the treated cells with a low level laser only (660 nm, 5 min) was assayed. 

### 2.5. The Statistical Analysis

Data were statistically described in terms of mean ± standard deviation (± SD) and standard error means (SEM). The comparison between the two study groups was done using a one-way analysis of variance (ANOVA) test with Bonferroni posthoc multiple two group comparisons. All statistical analyses were performed using a computer program IBM SPSS (Statistical Package for the Social Science; IBM Corp, Armonk, NY, USA) release 22 for Microsoft Windows. *P*-values of less than 0.05 were considered statistically significant.

## 3. Result and Discussion

### 3.1. FT-IR Spectroscopy

**Figure 1:**
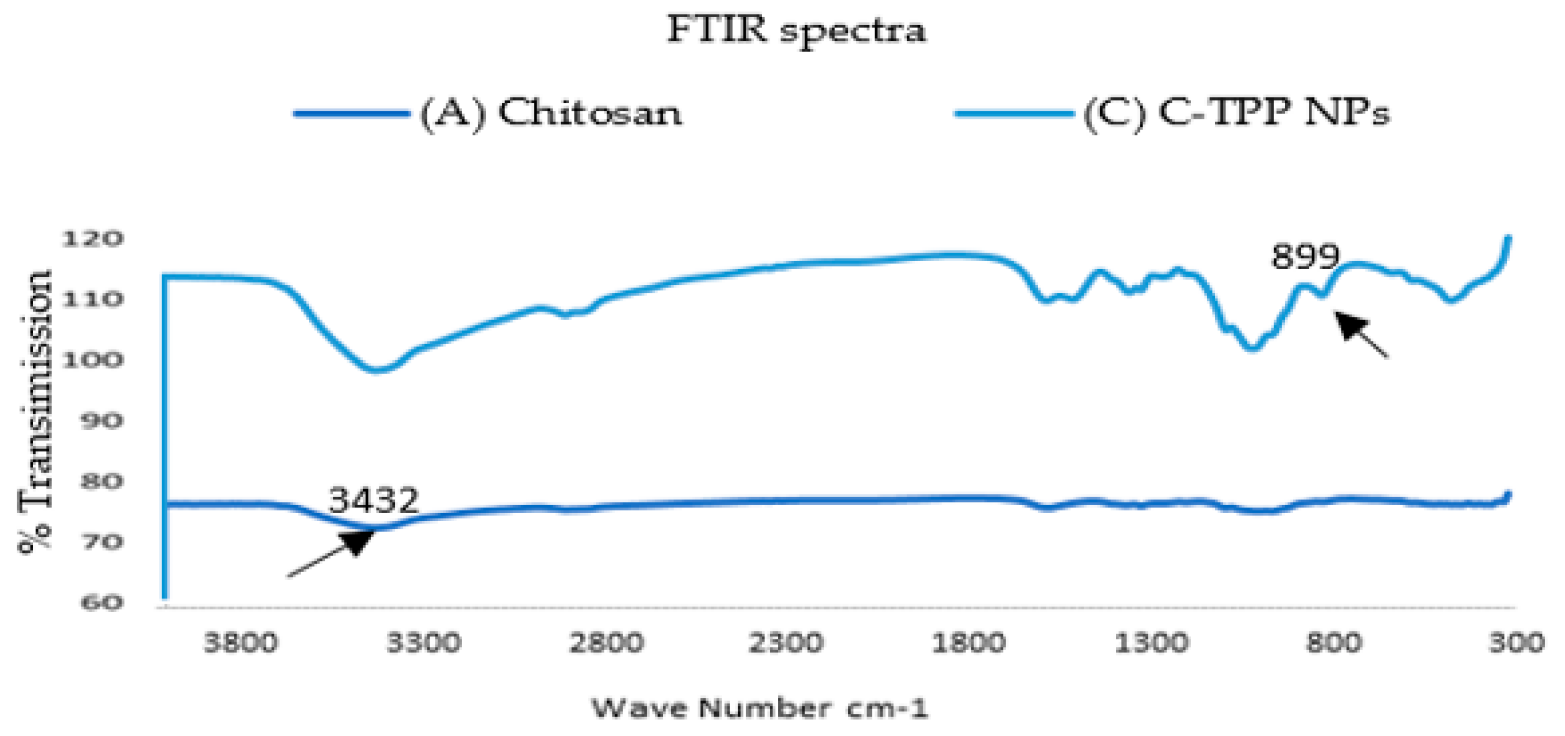
Fourier transform infrared (FT-IR) spectra for (**A**) Chitosan Extract, (**B**) C-TPP NPs.

As shown in (Figure 1), the FT-IR analysis of chitosan, and C-TPP NPs were carried out to determine the functional groups and consequently analyze the environment of capping ligands responsible for the stability of biosynthesized polymeric nanoparticles. For the chitosan, a strong band in the region (3432–524) cm^−1^ appeared. This region was corresponded to N-H and O-H stretching. The peak which appeared at 3432 cm^−1^ was attributed to the symmetric stretching vibration of OH. The peak at 2920 cm^−1^ referred to the symmetric and the asymmetric stretch of CH_3_ and CH_2_. The peak which appeared at 1631 cm^-1^ was attributed to C-O secondary amide stretch. The peak at 1382 cm^−1^ referred to δs(CH_3_) in the NHCOCH_3_ group. The peak around 1032 cm^−1^ was attributed to ʋ(C–O) in secondary OH group. All bands found in the spectra of chitosan were reported by Ibitoye et al. [14]. The FT-IR spectrum of C-TPP NPs revealed a peak at 3429 cm^−1^ which was attributed to the symmetric stretching vibration of OH. The peak at 2923 cm^−1^ referred to the CH_3_ symmetrical stretch and CH_2_ asymmetric stretch. 

The absorption peak that appeared at 2923.88 cm^−1^ indicates the presence of CH stretch. The peaks at (1636, 1412,1153 and 1323) cm^−1^ were attributed to secondary amide stretch, CH_2_ bending, CH_3_ symmetrical deformations and ʋs(C–O–C) (glyosidic linkage), respectively. C-TPP NPs spectrum shows new peaks at 899 cm^−1^ which was assigned to the characteristic bands of TPP polyions (P-OH and P=O stretches) [15]. The formulation of C-TPP NPs by the ionic gelation method (ionic crosslinking technology) is based on the formation of complexation between the positively charged amine group of chitosan and negatively charged polyions in tripolyphosphate (TPP) as reported in Aruna et al. [6] and Wang et al. [16].

### 3.2. Particle Size and Zeta Potential Measurements 

**Figure 2:**
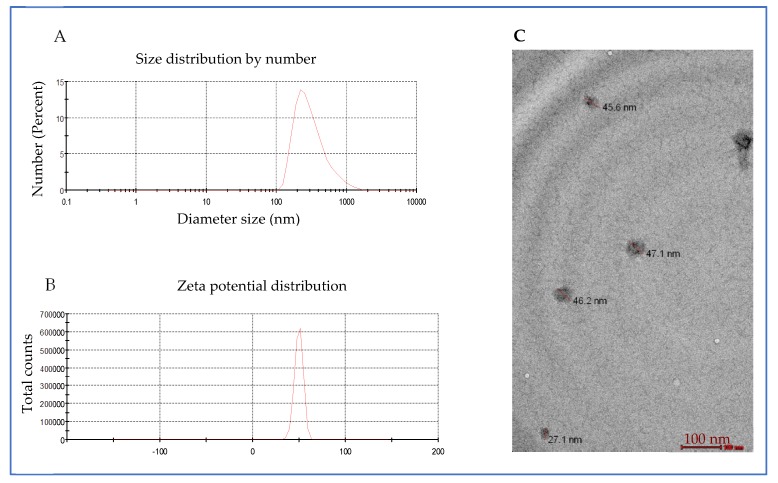
C-TPP NPs characterization (**A**) particle diameter size distribution, (**B**) Zeta Potential, (**C**) transmission electron microscope (TEM) images.

Aqueous solutions revealed broad diameter size distribution of C-TPP NPs from ~100 nm to ~1000 nm with a peak diameter size of 331 nm as measured by DLS as shown in (Figure 2A). Zeta potential (ZP) of the C-TPP NPs was 49.8 mV. ZP reflects the electrical superficial charge of the particles [17] and the dispersion stability [18]. Large values of zeta potentials predict a more stable dispersion. Gan et al. [17] reported that NPs that have ZP above 30 mV are stable in suspension because the surface charge prevents the particles aggregation.

### 3.3. Transmission Electron Microscope Characterization

TEM measurements of the C-TPP NPs appeared in (Figure 2C). The mean diameter size of C-TPP NPs measured was 75 nm (±50 nm) with spherical shape. The contour of C-TPP NPs is due to the high stability of the synthesized NPs [19]. The results show an increase in the diameter size measured by DLS compared to TEM. This increase is due to the light scattering, which leads to shifting the measured particles size towards larger values as reported by Souza et al. [20]. 

### 3.4. Viability Analysis and Cells Screening

**Figure 3:**
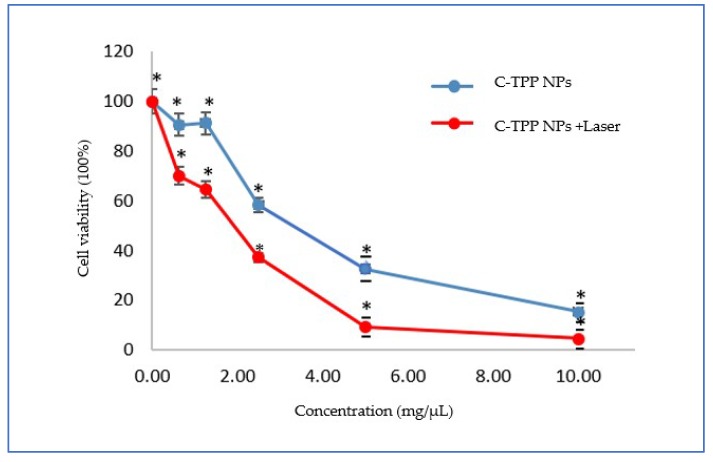
Cell viability assay on Caco2 cells versus the concentrations of C-TPP NPs, in the absence and in the presence of laser irradiation Data presented as mean ± SD. * *p* < 0.05 compared between the two groups.

The cell viability assay for Caco2 cells versus the numerous C-TPP NPs concentrations, in the absence and the presence of the NIR laser, as determined by the MTT assay showed in Figure 3. *p* < 0.05 was determined for all comparisons, the *p*-values less than 0.05 was considered statistically significant. The results appeared significant effect of C-TPP NPs against the Caco2 cells compared to control (untreated cells). 

**Figure 4:**
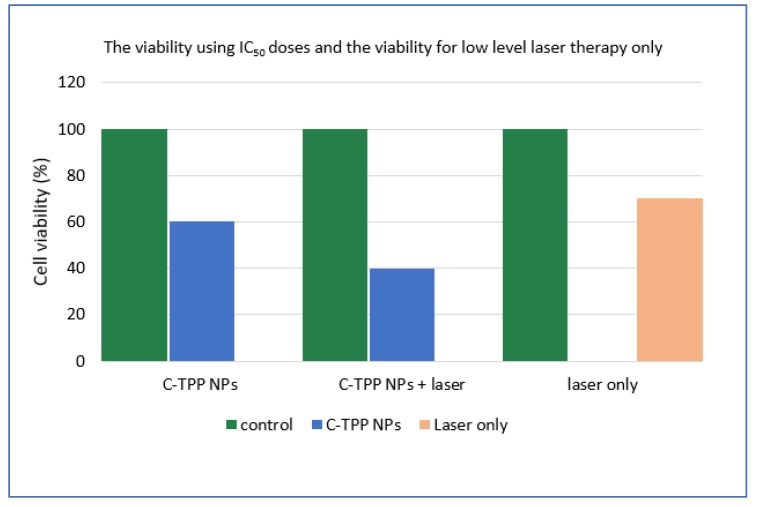
The activity of the C-TPP NPs against Caco 2 cells in the presence and the absence NIR. The cell viability against low level laser therapy only.

The IC_50_ dose (determined by GraphPad Prism 7 software) was 3 µg/mL in the dark experiment. In the case of laser exposure, the IC_50_ dose was 2 µg/mL. The IC_50_ dose decreased to two-thirds in the presence of laser. The cell viability assayed using the trypan blue method in the absence and the presence of NIR was significant compared to the control (untreated cells) as shown in (Figure 4). The cooperation between the NIR laser and the NPs causes dramatic change on the viability percentage. The viability of the exposed cells to laser decreased compared to non-exposed cells. On the other hand, the treatment with laser only was obviously insignificant on the cell viability compared to other treatment techniques ((NPs only) or (NPs plus laser)) as represented in (Figure 4). The previous observations finding agree with Wang et al. [21] who reported a decrease in the viability as well as the inhibition of the growth of cancerous cells when treated with chitosan. Also, the result agrees with Loutfy et al. [10] who assays the viability of chitosan NPs against the liver cancer cell line (HepG2). The results agree with Lai et al. [22] who found that the gathering between the NIR and NPs increases the efficiency of treatment drastically through the effective conversion of light energy to thermal.

**Figure 5:**
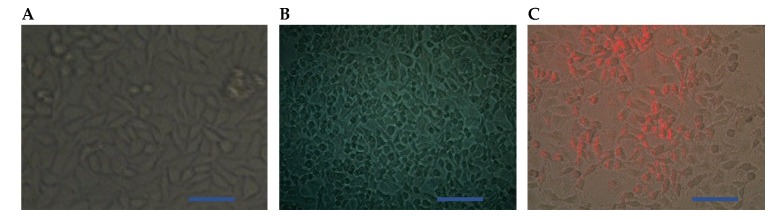
Representative photomicrographs (**A**) Control untreated cells (**B**) Cells treated with 3 µg/mL of C-TPP NPs, (**C**) Optical tomography (using NIR laser) for the treated cells (3 µg of C-TPP NPs), Scale bar 20 µm.

**Figure 6:**
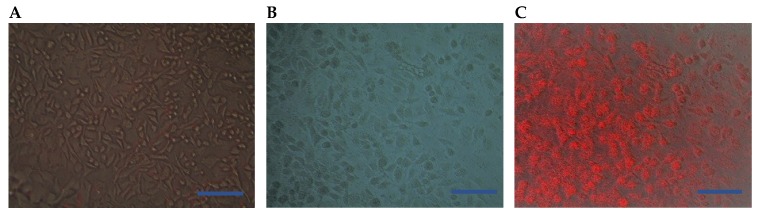
Representative photomicrographs (**A**) Optical tomography (using NIR laser) for Caco2 cells was treated with NIR laser only, (**B**) Cells treated with 2 µg/mL of C-TPP NPs, (**C**) Optical tomography using NIR laser for the treated cells (2 µg of C-TPP NPs), Scale bar 20 µm.

In the present study, the morphological changes for the treated cells (using the IC_50_ doses) was observed under the inverted microscope. The photomicrographs of the treated cells and the control (untreated cells) were displayed in (Figure 5 and Figure 6). The cells were photographed using a digital camera in the presence of NIR laser (Figure 5C and Figure 6A,C) and in the absence of NIR laser (Figure 5A,B and Figure 6B). The results revealed a strong effect of the C-TPP NPs. This effect appeared as shrinkage as well as the accumulation of dead Caco 2 cells compared to the control. On the other hand, the shining effect of the C-TPP NPs (Figure 5C and Figure 6C) appeared when the treated cells were exposed to NIR laser during cell imaging. This shining effect did not appear on the cells treated with NIR only (Figure 6A). Besides, no shining effect appeared on the photographed cells (Figure 5B and Figure 6B) because they were not exposed to NIR during the imaging. This result agrees with Yi et al. [23] who analyzes the significant effect of near-infrared fluorescent probes in cancer imaging and therapy. Additionally, Marpu et al. [7] who reported the usage of chitosan base in synthesizing various clinical imaging agents and its effect in photonic application. Furthermore, Ash et al. [5] reported the significant effect of the NIR laser in deep tissue imaging. The dominant tissue absorbents of NIR photons are limited to hemoglobin/ myoglobin, water, and lipids. This was attributed to the long wavelength of the NIR laser compared to UV laser and consequently the NIR does not damage normal cells.

The presented study avoids the utilization of all harmful materials encapsulated with nanoparticles for therapeutic and imaging purposes. Consequently, our composite is a suitable candidate for in vivo application in the preoperative localization of cancer as well as during intra-operative management, where the chitosan NPs are accumulated in the cancer tissue more than they do in the normal tissue due to the enhanced permeability and retention (EPR) effect [10].

## 4. Conclusions

In this study, a monodispersed, stable (ZP > 30 mV) chitosan-TPP was successfully prepared in nano form using ionic gelation methods. Anticancer activity of the synthesized NPs against Caco 2 cells was carried out by MTT assay. The needed amount of C-TPP to attain IC_50_ lowered to two-thirds in the laser presence. The shining effect of the C-TPP NPs appeared when the treated cells were exposed to NIR laser during the cell imaging. Future explorations are in progress to investigate the pharmacokinetics, systemic toxicity, photothermal efficiency, and molecular imaging of natural C-TPP NPs in vivo applications.
